# Dual Regulation of Ionic Effect on *Zostera marina* L. Seed Germination and Leaf Differentiation in Low-Salinity Conditions

**DOI:** 10.3390/plants14020254

**Published:** 2025-01-17

**Authors:** Peilong Li, Yaping Gao, Zengjie Jiang, Linjie Wang, Xiaoli Sun, Jiaqi Wang, Jing Wang, Haidong Sui, Junwei Wang, Yitao Zhang

**Affiliations:** 1 State Key Laboratory of Mariculture Biobreeding and Sustainable Goods, Yellow Sea Fisheries Research Institute, Chinese Academy of Fishery Sciences, Qingdao 266071, China; lipeilong666@hotmail.com (P.L.);; 2 Graduate School, Chinese Academy of Agricultural Sciences, Beijing 100081, China; 3 Laboratory for Marine Fisheries Science and Food Production Processes, Qingdao Marine Science and Technology Center, Qingdao 266237, China; 4 College of Life Science, Qingdao University, Qingdao 266071, China; 5 SEE Foundation, Beijing 100038, China; 6 Rongcheng Chudao Aquaculture Co., Ltd., Rongcheng 264312, China

**Keywords:** germination, leaf differentiation, osmotic pressure, biochemical traits, antioxidant enzymes

## Abstract

Low-salinity conditions are generally used in land-based cultivation to promote the germination and growth of *Zostera marina* L. and to improve the restoration effect of seagrass beds. Different salinity conditions lead to morphological and physiological differences. To investigate the impacts of salinity and osmotic pressure on the germination and early development of *Zostera marina* seeds, this study utilized seawater with different salinity conditions and PEG-6000 solutions to simulate various non-ionic osmotic pressures and examine the germination, cotyledon growth, and leaf differentiation over 28 days, as well as determine the biochemical traits on days 1, 3, 5, and 7. The results show that the cumulative germination rate in LS-0 was 91.6%, but it was not significantly affected by the PEG solutions. The different salinities (5, 10, and 15) had no significant effect on the germination rate, which ranged from 76.4% to 78.8%: low salinity and low osmotic pressure stimulated the germination by accelerating the water uptake through increased osmotic pressure differences. The leaf differentiation was regulated by the osmotic pressure and salinity. In LS-10, the most used condition, the leaf differentiation rate was 35.2%, while PEG-10 displayed 6.4%. The total soluble sugar and soluble protein in the seeds decreased. Antioxidant enzyme activities were activated under low-salinity conditions, which supported germination within a tolerable oxidative stress range.

## 1. Introduction

**Figure 1:**
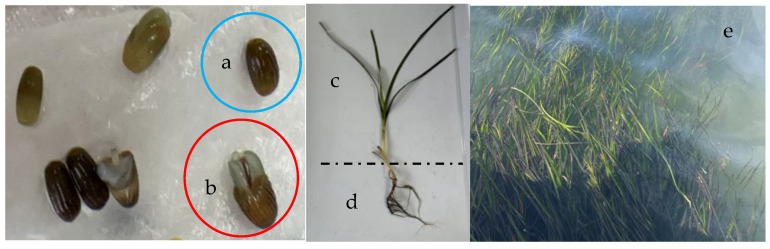
Seeds, adult shoot, and natural seagrass bed. (**a**) shows an ungerminated seed. (**b**) shows a germinated seed. (**c**) shows the vegetative shoots. (**d**) shows the rhizome and roots. (**e**) shows the condition of a natural seagrass bed in Sanggou Bay.

Seagrass beds are one of the most productive systems in ecosystems worldwide [1]. They provide essential food and habitat for marine life and contribute to ecological functions, such as mitigating ocean acidification [2,3]. However, due to multiple factors related to human activities and climate change, the area of seagrass beds is rapidly declining, resulting in a gradual loss of their ecological functions [4,5,6]. To restore coastal ecosystems, seagrass beds are mainly recovered via natural restoration, seed-based restoration, and direct transplantation. The effect of direct transplantation is more obvious. In past restoration works, the seagrass used for transplanting was sourced from existing seagrass beds, but improper excavation activities often threatened the survival of seedlings in both the donor and recipient seagrass beds [7]. In current restoration works, *Zostera marina* L. is one of the main species used to restore seagrass beds. Being the most widely distributed in the Northern Hemisphere, *Zostera marina* is commonly found in a mixing area of mud and sand as a substrate and it can reproduce both asexually through clonal growth and sexually through seeds [8]. *Zostera marina* seeds are normally black or dark brown; their size varies between regions, but they are usually no more than 4 mm in length and 2 mm in diameter [9] (Figure 1a,b). Adult *Zostera marina* consists of simple alternate leaves, rhizomes, and roots (Figure 1c,d). In recent years, the development of artificial cultivation techniques for *Zostera marina* has enabled the large-scale, land-based production of seedlings, which now form the basis of most restoration projects. Through short-term cultivation in a controlled environment, a substantial number of seedlings can be produced for transplantation [10,11]. Compared with traditional methods, land-based cultivation achieves a higher germination rate, preserves natural seagrass beds, and yields stronger seedlings with improved survival rates after transplantation [12]. To enhance the production efficiency, the germination of *Zostera marina* seeds is typically stimulated by reducing the seawater salinity, often using water with a salinity of 10 or even freshwater [13]. Seedlings from seeds germinated at different salinities, when transferred to the same salinity for cultivation, and seedlings from seeds germinated at the same salinity, when transferred to different salinities, show distinct developmental patterns [14,15]. These findings suggest that salinity fluctuations significantly influence the biochemical processes of *Zostera marina*, impacting both its morphology and physiology across various growth stages.

Seawater contains a large number of ions, including Na^+^, K^+^, Mg^2+^, Ca^2+^, Cl^−^, and SO_4_^2−^. In the oceanic environment, the ionic composition of seawater is relatively stable. However, due to the influence of climate, terrain, and other factors, freshwater or other sources of water in the offshore area have different impacts on the salinity and ion composition of offshore seawater [16,17], which can seasonally affect seagrass population characteristics in natural marine habitats [18,19]. Both natural salinity fluctuations in offshore waters and artificial salinity regulation in land-based cultivation influence the life cycle of *Zostera marina*. Changes in the salinity first affect the ionic composition and concentration in seawater, modifying the ionic interactions, and consequently, the osmotic pressure [20]. For plants, salinity fluctuations introduce salt stress, which has a negative impact on the growth and development. Salt stress leads to both osmotic pressure and ion stress, disrupting the balance between intracellular and extracellular osmotic pressures, as well as altering the water flow and cellular water content. Excessive Na⁺ may accumulate within the cells, and pathways for other ions may be similarly disrupted [21]. This imbalance impairs the cellular ion homeostasis [22], disturbing the biochemical processes, and ultimately, inhibiting growth [23]. Concurrently, plants face oxidative damage, which activates antioxidant enzymes. In response, plants regulate the ionic and osmotic homeostasis pathways; modulate the hormone signaling; adjust the cytoskeletal dynamics; and alter the cell wall composition [24,25], for example, by managing intracellular ion and organic solute concentrations or modifying cell wall elasticity [26]. Therefore, for the existing land-based cultivation process, changes in the ion composition can affect the growth and development of *Zostera marina* without significant changes in the salinity, as well as leading to a decrease in the yield.

The activation of *Zostera marina* seed germination under low-salinity conditions has been an important part of the consensus and practical production [27]. Hundreds of thousands of *Zostera marina* seedlings are cultivated and transplanted into the sea each year. Following an extensive evolutionary history, moving from marine to terrestrial environments and back to the sea, *Z. marina* has adapted to salinity conditions of approximately 30, becoming a dominant seagrass species [28]. However, reductions in salinity can still induce stress. Research on the effect of low salinity on the early development of *Zostera marina* primarily addressed the morphological differences in seeds and seedlings over extended periods [29,30]. However, there has been limited continuous observation of seed germination, internal biochemical responses under various salinity and ion-free osmotic pressure conditions, and subsequent seedling development. Additionally, the specific effects of ion-free osmotic conditions, distinct from seawater, on seed germination and growth remain unexplored [31]. And no findings can explain the reasons for the inconsistent survival rate of seedlings cultivated in different batches under the same salinity conditions.

In this study, common low-salinity conditions for germination activation were selected, and PEG-6000 solutions at various concentrations were used to simulate the corresponding ion-free osmotic pressure conditions. Seawater with a salinity of 30 was used as the control group. The effects of salinity and osmotic pressure on seed germination, cotyledon growth, and leaf differentiation were investigated through continuous observation. Additionally, the physiological changes and antioxidant enzyme activities in the seeds during germination were also measured. This research explored the responses of *Zostera marina* seeds to low-salinity and ion-free osmotic pressure conditions during germination and seedling development, with separate discussions on the effects of ions and osmotic pressure on the early development. The goal of this study was to investigate the separate and combined effects of salinity and non-ionic osmic pressure on the germination and early development of *Zoster marina* in order to explain the inconsistent seedling development in different cultivation batches under the same salinity conditions.

## 2. Materials and Methods

### 2.1. Experimental Materials

*Zostera marina* seeds were collected from Sanggou Bay, Rongcheng City, Shandong Province, in July 2023 (37°02′38.3″ N, 122°33′30.3″ E). The collection was carried out when mature seeds were visible in flame buds. During the collection, the flame buds with seeds were removed from the genital branches and placed in mesh bags, which were then securely tied and suspended in the natural sea area. The seeds in the bag were collected after they fell off naturally. The collected mature seeds were brought back to the laboratory in an insulated foam box with ice packs and stored in natural seawater with a salinity of 30. The seawater was refreshed every 3 days during storage. Low-salinity solutions were obtained by diluting seawater with deionized water. The LS-0 solution contained only deionized water.

### 2.2. Experimental Design

The experiment was conducted from October 2023 to May 2024 at the Yellow Sea Fisheries Research Institute (YSFRI) and was divided into two parts. The first part focused on the germination and seedling development of *Zostera marina* under different saline seawater conditions and various concentrations of PEG-6000 solutions over 28 days. In the second part, the biochemical traits of the *Zostera marina* seeds were examined under these same conditions for 7 days.

In the first part of the experiment, a total of 8 treatments were applied (Table 1). PEG-6000 solutions with osmolarities equivalent to salinities of 5, 10, and 15, but without ions, were prepared according to the formula by Burlyn et al. [32] (corresponding relationship: LS-5 to PEG-5, LS-10 to PEG-10, and LS-15 to PEG-15), with a salinity of 30 serving as the control group to present the growth condition in a natural environment. At the beginning of the experiment, each 100 g sample of *Zostera marina* seeds was placed in a sucrose solution (120 g sucrose in 200 mL deionized water) to separate the dense seeds, with the floating seeds discarded. The germinated seeds were removed. The seeds that were solid in texture, dark brown or black in color, undamaged, and consistent with the morphological characteristics of healthy seeds were manually selected. The selected seeds were then washed three times with sterile seawater, soaked in 2.5% NaClO solution for 3 min to sterilize their surface and minimize microbial contamination during the experiment to ensure that they remained free from fungal or bacterial growth during the germination process, and rinsed again in sterile seawater at least three times until the solution was clear.

Each treatment included five replicates, with 50 seeds per replicate. Each seed was placed individually in a 2 mL EP tube that contained 1.8 mL of the assigned solution, with all 50 tubes stored in a zip-lock bag (18 cm × 25 cm) in an illumination incubator. The inner surface of the incubator was covered with tin foil and the temperature was maintained at 20 °C in darkness for the entire 28-day observation period.

The second part of the experiment used the same treatments and culture conditions as the first. Each treatment had three replicates, with an average of 150 seeds per replicate, which were used to measure the biochemical traits (total soluble sugar content, total soluble protein content, MDA content, CAT activity, and SOD activity). Samples were collected on days 1, 3, 5, and 7. After each sampling, the seeds were blotted dry with absorbent paper, placed in 2 mL cryotubes, quickly frozen in liquid nitrogen for 5 min, and stored at −80 °C.

### 2.3. Sample Collection and Determination

After thawing, the samples were blotted dry with absorbent paper, and approximately 0.1 g of each sample was weighed and ground to a homogenate using an automatic sample grinder, which had been pre-cooled for 20 min. Subsequent procedures followed the protocols provided by the assay kits: total soluble sugar (Beijing Solarbio Science & Technology Co., Beijing, China), total soluble protein (Nanjing Jiancheng Co., Nanjing, China), MDA (Nanjing Jiancheng Co.), CAT activity (Nanjing Jiancheng Co.), and SOD activity (Nanjing Jiancheng Co.). The salinity was measured using a Multi 3630 IDS (WTW, Munich, Germany). The samples were added to a 96-well plate and measured with a Multiskan FC microplate reader (Thermo Fisher Scientific, Waltham, MA, USA).

### 2.4. Morphological Analysis

The numbers of germinated seeds and developed seedlings were recorded, and various indices were calculated, including the germination rate, mean time of germination (MTG), germination index (GI), germination energy (GE), cotyledon elongation, and leaf differentiation rate. Seed germination was identified by signs such as seed coat rupture, seed cracking, and cotyledon extension. The calculation formulas were as follows:

(1) Germination rate (%) = (number of germination seeds/number of test seeds) × 100%.

(2) Mean time of germination (MTG) (d) = ∑(n_i_ × d)/N.

Here, n_i_ represents the number of seeds germinated on day i, d represents day i of the germination statistics, and N is the total number of germinated seeds.

(3) Germination index (GI) = ∑(Gt/Dt).

Here, Dt represents the number of germination days, and Gt represents the number of germinated grains on the days corresponding to Dt.

(4) Germination energy (GE) = (the number of seeds germinated at the peak of germination per day/number of test seeds) × 100%.

(5) Peak value (PV) = number of germinated seeds at germination peak/corresponding germinating days.

(6) Germination value (GV) = average germination rate × peak value.

(7) Cotyledon elongation: the length of the cotyledon was divided into three intervals (<1 cm, 1–3 cm, >3 cm), and the proportion of the number of cotyledons in the interval to the number of seeds tested was calculated.

(8) Leaf differentiation rate (%) = (the number of seedlings that differentiated leaves/number of test seeds) × 100%.

### 2.5. Statistical Analysis

All statistical analyses were performed using Excel 2021 and GraphPad Prism 9.0. A one-way ANOVA and two-way ANOVA were performed where required.

## 3. Results

### 3.1. Effects of Salinity and Osmotic Pressure on Seed Germination

In the first 3 days of treatment with different salinity levels and PEG-6000 solutions, the germination rate increased significantly across all the conditions. The germination rate in LS-0 increased the fastest, reaching 81.6% at 72 h. The rates in LS-5 and LS-10 showed similar trends, reaching 65.6% and 68%, respectively. Over the 28-day germination experiment, the increase in LS-0 slowed after the third day but remained the highest among the treatments, reaching 91.6%. The germination rate in LS-5 and LS-10 followed a consistent trend, slowing down after day 6. The rate in LS-15 reached 72% by day 12 and then stabilized. Additionally, the germination trends in LS-5, LS-10, and LS-15 began to converge from day 10 onward, with the final germination rates on day 28 of 76.4%, 76.5%, and 78.8%, respectively. The control group, LS-30, showed only a 29.6% germination rate by day 28 but maintained an upward trend.

**Figure 2:**
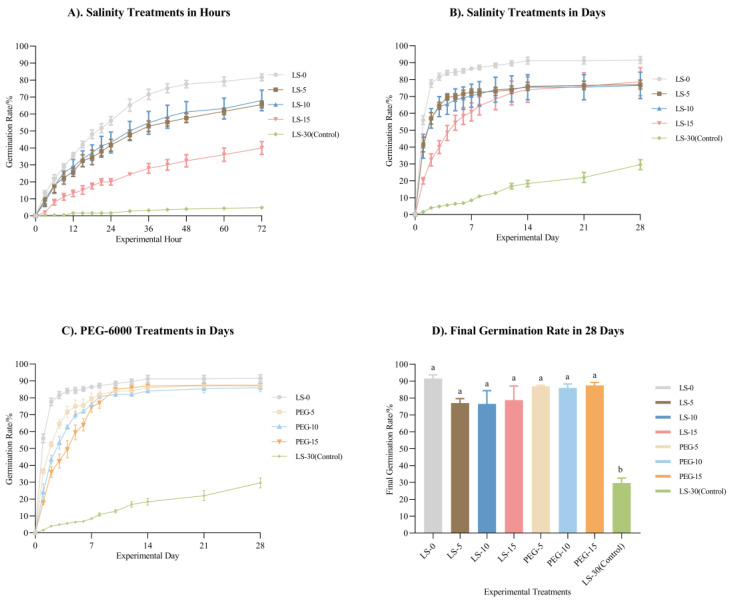
The cumulative germination rates over 28 days (mean ± SEM). (**A**) shows the germination rates of ungerminated seeds in the different salinity treatments for the first 72 h. (**B**) shows the germination rates of ungerminated seeds in different salinities for 28 days. (**C**) shows the germination rates of ungerminated seeds in different concentrations of PEG-6000 solutions for 28 days. (**D**) shows the final germination rates of germinated seeds treated with seawater with different salinity levels and PEG-6000 solutions with different concentrations. Different letters indicate a significant difference in each parameter between the different solution treatments after the 28-day experiment (*p* < 0.05).

Figure 2C shows the germination rates under different osmotic pressure treatments (LS-0, PEG-5, PEG-10, and PEG-15), ranked from fast to slow. On days 5 to 7, the germination rates began to decelerate, with convergence occurring around days 9 to 11. By day 28, the germination rates of all osmotic pressure treatments ranged from 86% to 90.4%. A two-way ANOVA revealed an interactive effect between the days and treatments (*p* < 0.0001), with both factors showing a significant main effect on the germination rate (*p* < 0.0001 and *p* < 0.0001, respectively). A one-way ANOVA showed that low-salinity or low-osmotic conditions had a significant effect on promoting the germination rate.

As shown in Table 2, the mean time of germination (MTG) increased with rising ion concentrations and osmotic pressure across the different treatments, from 2.02 to over 28. Except for LS-30, the MTG values in LS-15 and PEG-15 were 2.14 and 2.00 times higher than those in LS-0, respectively. The germination index (GI), germination energy (GE), peak value (PV), and germination value (GV) decreased as the salinity or osmotic pressure increased.

### 3.2. Effects of Salinity and Osmotic Pressure on Seedling Growth

In the first week, the cotyledon growth in the LS-0 treatment was significantly faster than that in the other treatments, but no individual cotyledon exceeded 3 cm. Meanwhile, in all the treatments, except for LS-10, the cotyledon growth was generally less than 1 cm, which accounted for 9.3–64% of all the individuals. Except for LS-0, all the treatments had individual cotyledons elongated beyond 3 cm. From the second to the fourth weeks, because of the continuous germination of the seeds, the proportions of the different cotyledon lengths fluctuated. In LS-10 and LS-15 in the fourth week, the majority of cotyledon reached over 3 cm, which accounted for 42.4% and 46.0%, respectively, while most cotyledons in the other treatments were still less than 1 cm. After the first week, there was no significant change in the development of the cotyledons in LS-0, with most remaining under 1 cm (Table 3).

Over the 28-day period, no individuals in any treatments exhibited leaf differentiation within the first 7 days. Leaf differentiation in LS-5, LS-10, and LS-15 began on day 7, with LS-10 and LS-15 showing more consistent and higher rates than LS-5 from day 7 to day 28, reaching 35.2%, 40%, and 16.4%, respectively, on day 28. In LS-0, no leaf differentiation was observed throughout the 28 days. LS-30 exhibited the lowest leaf differentiation rate, reaching only 1.3% by day 28.

**Figure 3:**
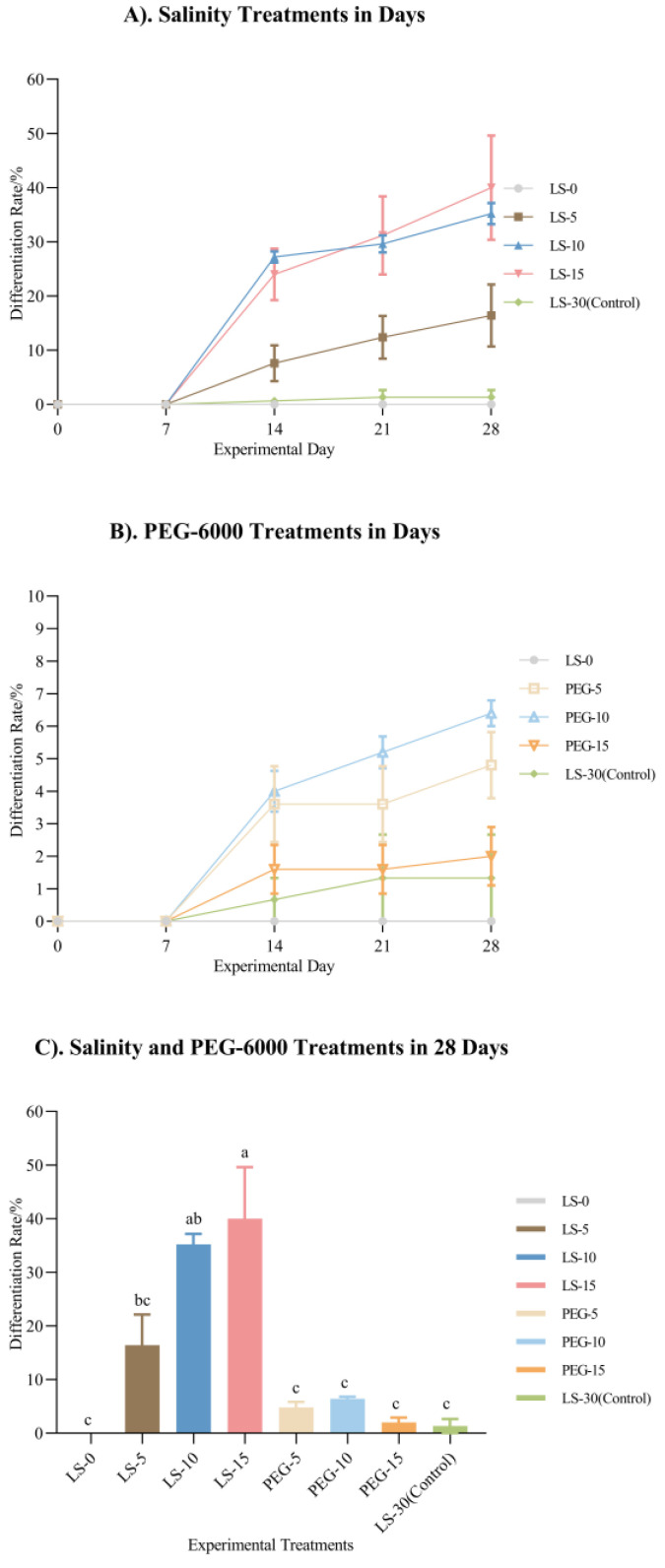
Leaf differentiation rates of 28-day *Zostera marina* seedlings (mean ± SEM). (**A**) shows the leaf differentiation rates of ungerminated seeds treated with different ion concentrations. (**B**) shows the leaf differentiation rates of ungerminated seeds treated with different concentrations of PEG-6000 solution. (**C**) shows the final leaf differentiation rates of each treatment after 28 days. Different letters indicate a significant difference in each parameter between the different solution treatments after the 28-day experiment (*p* < 0.05).

For the PEG treatments, no leaf differentiation occurred by day 7. The leaf differentiation began in the PEG treatments (except for in LS-0) after day 7, though at a generally low level. A two-way ANOVA revealed an interactive effect between the days and treatments (*p* < 0.0001), with both factors showing a significant main effect on the leaf differentiation rate (*p* < 0.0001 and *p* < 0.0001, respectively) (Figure 3).

### 3.3. Effects of Salinity and Osmotic Pressure on Biochemical Traits

During the 7-day germination period, the soluble sugar content in the *Zostera marina* seeds exhibited a fluctuating downward trend across the treatments, except for LS-30, where the trend remained relatively stable. Specifically, the LS-10 and PEG-10 treatments showed rapid decreases from 57.24 mg/g and 59.14 mg/g to 32.85 mg/g and 19.72 mg/g, respectively, followed by gradual increases to 42.97 mg/g and 40.03 mg/g. In contrast, the LS-15 and PEG-15 treatments displayed gradual declines from day 0 to day 5 and then slowly rose from day 5 to day 7.

In terms of the soluble protein content, all treatments, except for LS-30, showed general decreases. For LS-0, after entering the germination stage, the overall level remained low, from 10.28 mg/g to 8.62 mg/g. For both the ionic concentration and osmotic pressure treatments, the soluble protein content showed a moderate declining trend with a gradient decrease correlating with a higher salinity or osmotic pressure from day 1 to day 7.

Except for LS-30, the MDA content in all the other treatments showed a relatively rapid decline. Among them, LS-0 exhibited the fastest decrease, where it dropped from 83.90 nmol/g to 6.52 nmol/g. The MDA reduction rate corresponded to increases in the salinity and osmotic pressure, with similar distribution patterns observed on days 5 and 7.

The CAT activity in the different salinity and osmotic pressure treatments increased slightly on the first day and then decreased gradually from days 1 to 5, followed by rapid increases from days 5 to 7. The degrees of increase were inversely related to the salinity and osmotic pressure. Specifically, the CAT activity in LS-0 rose by 5.14 times from day 5 to day 7, while in LS-15 and PEG-15, it increased by 3.12 times and 1.47 times, respectively.

**Figure 4:**
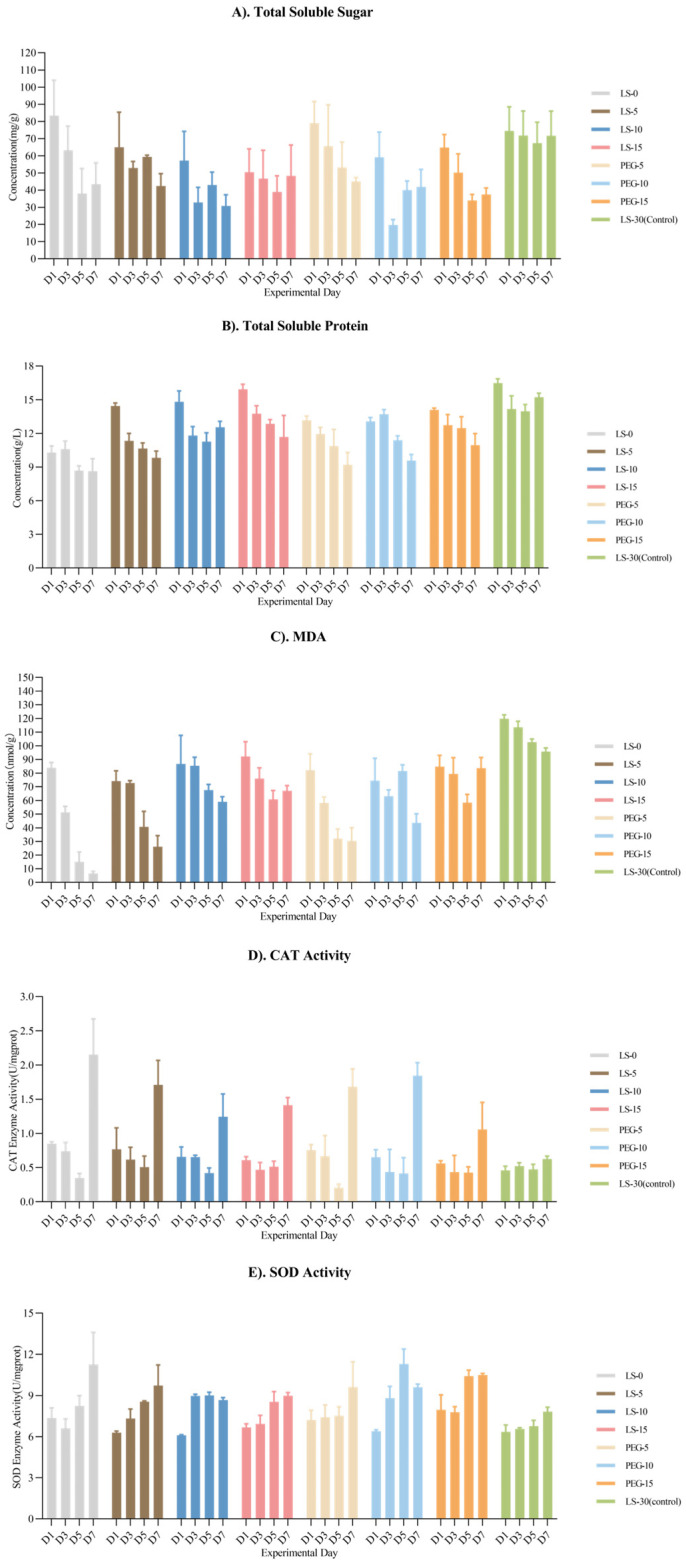
Total soluble sugar content, total soluble protein content, MDA content, CAT activity, and SOD activity in seeds during 7-day germination experiment. Mean ± SEM.

The SOD activity exhibited a fluctuating, time-dependent increase. In PEG-10, the SOD activity increased at the fastest rate until day 5; however, by day 7, it had decreased in both PEG-10 and PEG-5, while it remained steady in PEG-15. On the 7th day, LS-0 had the highest SOD activity, which was 1.67 times the initial value, while that of the other treatments ranged from 1.28 to 1.55 times the initial levels (Figure 4).

## 4. Discussion

### 4.1. Effects of Different Salinity Levels and Osmotic Pressures on Seed Germination

Water absorption is a critical step in seed germination for various plants [33]. Differences in ionic concentrations and compositions create distinct osmotic pressure conditions. Therefore, during the peak germination period within the first 72 h, we increased the observation frequency for germination rates in the salinity treatment groups. This allowed us to determine whether the seed germination process under different salinities exhibited distinct stages of water absorption and germination. The germination rate in LS-0 was higher than that in the other treatments and was significantly different from that in LS-15 and LS-30 (*p* = 0.0002 and *p* < 0.0001). The germination conditions in LS-5 and LS-10 were similar and higher than those in LS-15 and LS-30; this indicates that osmotic pressure was not the only factor that affected the germination and that ionic action and the initial state of the seeds may have affected the germination process.

Over the 28-day germination period, LS-0 consistently showed a higher germination rate than the other treatments, while the final germination rates of LS-5, LS-10, and LS-15 tended to converge, indicating that the moderate variations in salinity did not significantly impact the total germination rates. Furthermore, the ion presence appeared to inhibit the germination. This finding aligns with Orth et al.’s conclusion that the ion concentration has a limited influence on the cumulative germination rate [34]. Similarly, in Park et al.’s [35] results regarding the effect of salinity on seed germination, it was also shown that the germination rate at 0 salinity was about 20% higher than that at 15 salinity and about 40% higher than that at 30 salinity, while in our experiments, the germination rate at 0 salinity was 12.8% higher than that at 15 salinity and 62% higher than that at 30 salinity. The same decreasing trend in the germination rates from low salinity to high salinity was also observed in a species closely related to *Zostera marina*, *Zostera japonica* [36]. The germination rate in LS-30 continued to increase throughout the experiment, suggesting that in the absence of factors such as mold contamination [37] or low dissolved oxygen, LS-30’s cumulative germination rate might have eventually matched that of the lower-salinity treatments.

Under various osmotic pressure conditions, the *Zostera marina* seeds exhibited lower germination speeds as the osmotic pressure increased. This may reflect the seeds’ natural exposure to higher osmotic conditions, affecting the water absorption rates due to osmotic differences inside and outside the seeds. However, after 28 days, the total germination rates across the different osmotic treatments converged, suggesting that the osmotic pressure did not influence the cumulative germination rate in this experiment.

### 4.2. Effects of Different Salinity Levels and Osmotic Pressures on Seedling Growth

During the germination, the germination began from the water absorption rupture of the seed, and the cotyledon emerged from the middle rupture of the seed and gradually extended. Under natural conditions, the cotyledon grows above ground, later developing into vital organs, such as leaves and stems [38]. Thus, proper cotyledon development is essential for survival. Different treatments yielded different cotyledon growth trends. Although LS-0 displayed robust growth in the first week, further development did not occur. LS-5 showed better growth than LS-0 but was outperformed by LS-10 and LS-15, suggesting that moderate salinity may favor cotyledon growth. Similarly, the PEG-5 and PEG-10 treatments allowed for some growth over the 28 days, though the performance was still below that of the equivalent salinity treatments.

Leaf differentiation, a key factor in early seedling establishment, enables photosynthesis and influences various biochemical reactions [39,40]. In the 28-day experiment, leaf differentiation occurred in all salinity treatments except for LS-0, with LS-10 and LS-15 showing higher differentiation rates. In the presence of ions, lower salinity levels, such as LS-10 and LS-15, exhibited a strong difference from LS-30 in the leaf differentiation rates. The main reason for this might be the difference in the seed germination speed. Under the low-salinity conditions, the seeds germinated rapidly in the early stage and entered the subsequent development process. However, under the condition of 30 salinity, the natural condition, the seed germination process was not rapidly activated, and a slow germination state was maintained. Although LS-0 showed a similar trend to LS-30, the underlying reasons differed. The solution used in the LS-0 treatment group was deionized water, and the condition was far different from that of the natural sea area. At the same time, the experiment showed that LS-0 could effectively stimulate seed germination but was not conducive to seedling growth. Adams and Bates found that the greatest wet mass and rhizome length attained by *Zostera capensis* were 2.37 g/shoot and 208 mm/shoot at 15 salinity under laboratory-cultured conditions, and these values were 1.5 and 1.4 times higher than those at 35 salinity and 3.4 times and 1.6 times higher than those at 0 salinity [41]. In Deng et al.’s experiment on seedling establishment in a natural sea area, the highest seedling establishment rate was 55% [42], while in our results, the rate was 40 ± 9.61% in LS-15. This shows that in LS-15, the inventory situation was close to that under natural conditions. Leaf differentiation also occurred in all osmotic treatments but was less extensive than in the salinity treatments at the same osmotic level.

Thus, cotyledon growth and leaf differentiation in *Zostera marina* were influenced by both the salinity and osmotic conditions, with salinity exerting a more dominant effect. Different ion concentrations and osmotic pressures likely impacted the seed water absorption rates, and in LS-0, excessive pressure differences led to a rapid osmotic imbalance and over-absorption. This may have caused internal protein or membrane damage that hindered repair, which resulted in protein misfolding or dysfunction, thereby impairing subsequent growth [43]. After a certain osmotic pressure environment is applied, the pressure difference between the inside and outside of the seeds is reduced, and the imbibition and damage of the seeds during germination can be controlled, so the seeds still have the possibility to develop further. Additionally, certain ions, such as plumbum and chromium, can hinder plant growth [44], while others, such as iron at appropriate concentrations, can promote it [45]. Different ions have complex reactions with plant cells [46]. Several ions in seawater may act as catalysts or participate in some key reactions in the growth of *Zostera marina* with unknown functions [47].

### 4.3. Effects of Different Salinity Levels and Osmotic Pressures on Biochemical Traits

Soluble sugars play a critical role in providing energy, signaling, and stabilizing cellular structures in plant cells [48]. After 7 days of germination under different salinity and osmotic pressure conditions, the soluble sugar content showed various degrees of decrease, likely reflecting differences in the respiration and metabolic rates due to starch hydrolysis and changes in the intracellular solute content during water absorption [49,50]. Especially in LS-0, the solute sugar content decreased from 83.35 mg/g to 37.94 mg/g from day 1 to day 5. Including the low-salinity conditions, the overall decreasing trend in the soluble sugar content is consistent with the conclusion of Sugiura et al. [51], who also found rapid consumption. Tinna and Morten also found that sucrose concentrations decreased under low-salinity conditions [52]. Through transcriptome and metabolome analyses, Zhu et al. confirmed that great changes occurred in the internal energy metabolism process, such as in the tricarboxylic acid cycle during seed development to provide energy for germination [53]. The soluble sugar content increased in the four treatment groups at the late stage of germination, indicating that the germinated seeds gradually adapted to the osmotic pressure level in the environment and began to maintain cell homeostasis, while LS-0 continued to exhibit osmotic imbalance. The reduction in the soluble protein content may have been due to proteolytic activity converting proteins into peptides and amino acids in order to fuel germination or to synthesize other forms of proteins, such as membrane proteins, for osmotic regulation and cellular stability. The more rapid decline in LS-0 suggests that subsequent protein synthesis was inhibited, reflecting the severe osmotic and salinity imbalances [54,55].

CAT and SOD activities, along with MDA content, indicate oxidative stress levels during seed germination [56]. The rapid enzyme activity increases indicate heightened physiological activity and antioxidant responses, helping the seeds adapt to the environment. The decline in MDA content suggests that MDA was effectively eliminated as the antioxidant enzyme activity increased, maintaining oxidative stress within manageable limits [57]. Different treatments elicited various responses to oxidative stress caused by salinity. However, the complex antioxidant systems of plants require further study to clarify the differential effects on CAT and SOD activities under various salinity and osmotic pressure conditions [58].

## 5. Conclusions

The seeds in non-ionic solutions showed a higher cumulative germination rate, though the leaf differentiation was minimal or absent. Conversely, the seeds in ionic solutions had lower cumulative germination rates than those in non-ionic environments, yet they exhibited higher leaf differentiation rates. During the germination, the seeds consumed total soluble sugars and proteins, while the antioxidant enzyme activity significantly increased. This suggests that in the low-salinity and low-osmotic-pressure environments, the seed activity was triggered despite the oxidative stress, but within a tolerable range. The low salinity disrupted the seed dormancy, initiating germination by increasing the osmotic pressure difference across the seed membrane, thereby accelerating the water uptake essential for metabolic processes. However, this osmotic pressure difference required the seeds to expend more energy to maintain a cellular osmotic balance, impacting both the germination and seedling growth of *Zostera marina*.

Combining the different effects of different types of ions on different stages of plant growth, these findings highlight the importance of maintaining a stable ionic composition in water sources for land-based *Zostera marina* cultivation, where the pre-treatment and monitoring of water sources may be necessary to optimize the growth conditions. Further investigation is needed to fully understand how specific ions in low-salinity environments impact the growth and development of *Zostera marina* and to elucidate the physiological conversion processes of active substances involved in osmotic pressure regulation.

## Figures and Tables

**Table 1:** Experimental treatment designs.

Treatment	Condition
LS-0	Salinity 0, 20 °C, dark
LS-5	Salinity 5, 20 °C, dark
LS-10	Salinity 10, 20 °C, dark
LS-15	Salinity 15, 20 °C, dark
PEG-5	160.65 g PEG-6000/1 kg H_2_O, 20 °C, dark
PEG-10	239.00 g PEG-6000/1 kg H_2_O, 20 °C, dark
PEG-15	299.11 g PEG-6000/1 kg H_2_O, 20 °C, dark
LS-30 (control)	Salinity 30, 20 °C, dark

**Table 2:** Effects of salinity and PEG-6000 treatments on the germination of *Zostera marina*. Mean ± SEM. Different letters indicate a significant difference in each parameter between different solution treatments after the 28-day experiment (*p* < 0.05).

Treatment	MTG	GI	GE	PV	GV
LS-0	2.0 ± 0.2 ^c^	34.8 ± 0.9 ^a^	56.0 ± 2.5% ^a^	28.0 ± 1.3 ^a^	25.7 ± 1.4 ^a^
LS-5	2.0 ± 0.3 ^c^	27.0 ± 1.2 ^b^	41.6 ± 3.4% ^b^	20.8 ± 1.7 ^ab^	16.0 ± 1.7 ^b^
LS-10	2.1 ± 0.4 ^bc^	26.4 ± 3.2 ^b^	41.0 ± 6.7% ^b^	19.1 ± 4.7 ^b^	15.6 ± 4.2 ^b^
LS-15	4.3 ± 0.7 ^a^	17.3 ± 1.4 ^c^	20.0 ± 1.9% ^cd^	5.8 ± 0.7 ^c^	4.4 ± 0.4 ^b^
PEG-5	2.7 ± 0.2 ^abc^	26.2 ± 0.1 ^b^	36.5 ± 1.7% ^bc^	18.3 ± 0.9 ^b^	15.9 ± 2.8 ^b^
PEG-10	3.3 ± 0.5 ^abc^	21.3 ± 1.6 ^bc^	27.3 ± 1.8% ^bcd^	11.7 ± 2.8 ^bc^	9.9 ± 2.3 ^bc^
PEG-15	3.9 ± 0.2 ^abc^	18.2 ± 0.9 ^c^	21.5 ± 1.5% ^cd^	7.8 ± 1.0 ^c^	6.8 ± 0.9 ^c^
LS-30	4.3 ± 0.6 ^a^	3.4 ± 0.6 ^d^	3.2 ± 0.5% ^e^	1.1 ± 0.5 ^d^	0.6 ± 0.2 ^d^

**Table 3:** Effects of salinity and PEG-6000 treatments on growth of *Zostera marina* seedlings.

Time	Treatment	<1 cm	1–3 cm	>3 cm
Week 1	LS-0	60.4 ± 4.3% ^a^	26.0 ± 4.1% ^ab^	0 ^c^
	LS-5	49.6 ± 5.7% ^ab^	19.6 ± 4.3% ^c^	2.0 ± 1.3% ^c^
	LS-10	21.6 ± 5.7% ^c^	40.4 ± 4.7% ^a^	7.2 ± 0.6% ^ac^
	LS-15	35.6 ± 4.6% ^bc^	24.0 ± 1.3% ^ab^	1.6 ± 0.4% ^c^
	PEG-5	53.0 ± 3.7% ^ab^	22.8 ± 4.2% ^c^	3.6 ± 1.6% ^c^
	PEG-10	52.7 ± 4.7% ^ab^	23.2 ± 4.2% ^bc^	1.2 ± 0.8% ^c^
	PEG-15	44.0 ± 2% ^ab^	7.6 ± 1.3% ^c^	0.4 ± 0.4% ^b^
	LS-30 (control)	9.3 ± 1.3% ^d^	1.3 ± 0.7% ^d^	0 ^c^
Week 2	LS-0	60.8 ± 1.0% ^a^	28.4 ± 2.5% ^a^	2.0 ± 0.6% ^d^
	LS-5	46.0 ± 8.4% ^b^	15.2 ± 2.0% ^bc^	17.2 ± 6.7% ^bc^
	LS-10	20.8 ± 3.6% ^c^	16.8 ± 3.4% ^b^	38.8 ± 3.1% ^a^
	LS-15	17.6 ± 2.4% ^c^	12.8 ± 2.2% ^bc^	35.6 ± 7.9% ^ab^
	PEG-5	50.0 ± 1.8% ^ab^	12.8 ± 1.7% ^bc^	24.4 ± 1.9% ^abc^
	PEG-10	42.7 ± 3.5% ^b^	13.2 ± 2.1% ^bc^	29.6 ± 2.6% ^ab^
	PEG-15	57.6 ± 3.2% ^ab^	6.0 ± 1.3% ^c^	7.2 ± 1.0% ^c^
	LS-30 (control)	12.6 ± 2.7% ^c^	1.3 ± 1.3% ^d^	3.3 ± 1.3% ^c^
Week 3	LS-0	54.8 ± 2.7% ^ab^	34.4 ± 2.6% ^a^	2.0 ± 0.6% ^d^
	LS-5	37.6 ± 9.0% ^bc^	14.0 ± 3.1% ^b^	20.8 ± 6.8% ^bc^
	LS-10	19.6 ± 3.9% ^cd^	14.4 ± 3.5% ^b^	42.4 ± 2.5% ^a^
	LS-15	13.6 ± 1.0% ^d^	8.4 ± 2.3% ^b^	44 ± 8.3% ^a^
	PEG-5	44.5 ± 4.9% ^ab^	16.8 ± 2.8% ^b^	24.8 ± 2.1% ^abc^
	PEG-10	40.0 ± 3.1% ^ab^	9.2 ± 1.4% ^b^	35.6 ± 4.4% ^ab^
	PEG-15	60.8 ± 3.9% ^a^	5.6 ± 1.0% ^b^	8.8 ± 2.1% ^c^
	LS-30 (control)	7.3 ± 2.9% ^d^	2.6 ± 1.8% ^c^	4.0 ± 2.0% ^c^
Week 4	LS-0	53.6 ± 3.4% ^ab^	35.2 ± 2.8% ^a^	2.8 ± 1.0% ^d^
	LS-5	40.8 ± 9.4% ^abc^	10.0 ± 2.6% ^b^	19.2 ± 4.8% ^bc^
	LS-10	20.0 ± 3.9% ^cd^	14.8 ± 2.6% ^b^	42.4 ± 3.1% ^a^
	LS-15	14.8 ± 1.6% ^d^	8.4 ± 1.8% ^b^	46.0 ± 9.5% ^a^
	PEG-5	44.0 ± 5.2% ^ab^	16.0 ± 2.1% ^b^	26.4 ± 2.6% ^abc^
	PEG-10	34.7 ± 4.7% ^bcd^	14.8 ± 1.9% ^b^	34.8 ± 3.3% ^ab^
	PEG-15	62.8 ± 3.9% ^a^	8.4 ± 1.7% ^b^	9.6 ± 1.9% ^c^
	LS-30 (control)	13.3 ± 1.8% ^d^	8.0 ± 3.5% ^b^	4.7 ± 2.7% ^c^

Mean ± SEM. Different letters indicate a significant difference in each parameter between different solution treatments after the 28-day experiment (*p* < 0.05). The color-coded indicates that the cotyledon growth level had the highest proportion in each treatment.

## Data Availability

The data presented in this study are available upon reasonable request.
